# The Impact of Quercetin and Its Methylated Derivatives 3-o-Methylquercetin and Rhamnazin in Lipopolysaccharide-Induced Inflammation in Porcine Intestinal Cells

**DOI:** 10.3390/antiox11071265

**Published:** 2022-06-27

**Authors:** Zita Karancsi, Dóra Kovács, Nikolett Palkovicsné Pézsa, Péter Gálfi, Ákos Jerzsele, Orsolya Farkas

**Affiliations:** Pharmacology and Toxicology Department, University of Veterinary Medicine, 1078 Budapest, Hungary; karancsi.zita@univet.hu (Z.K.); kovacs.dora@univet.hu (D.K.); palkovicsne.pezsa.nikolett@univet.hu (N.P.P.); galfi.peter@univet.hu (P.G.); jerzsele.akos@univet.hu (Á.J.)

**Keywords:** oxidative stress, antioxidant, lipopolysaccharide, IPEC-J2 cells, interleukin-6, epithelial permeability, quercetin, 3-o-methylquercetin, rhamnazin, pig

## Abstract

Oxidative stress in the small intestine can lead to inflammation and barrier malfunction. The present study describes the effect of quercetin (Q), 3-o-methylquercetin (QM), and rhamnazin (R) on cell viability, paracellular permeability, production of intracellular reactive oxygen species (ROS), extracellular hydrogen peroxide (H_2_O_2_), and interleukin-6 (IL-6) after challenging jejunal cells (IPEC-J2) with different types (*Salmonella enterica* ser. Typhimurium, *Escherichia coli* O111:B4, and *E. coli* O127:B8) of lipopolysaccharides (LPS) applied in 10 µg/mL concentration. The intracellular ROS level increased after all LPS treatments, which could be decreased by all tested flavonoid compounds in 50 µM concentration. Extracellular H_2_O_2_ production significantly increased after Q and R treatment (50 µM). *S*. Typhimurium LPS could significantly increase IL-6 production of enterocytes, which could be alleviated by Q, QM, and R (50 µM) as well. Using fluorescein isothiocyanate dextran (FD4) tracer dye, we could demonstrate that *S*. Typhimurium LPS significantly increased the permeability of the cell layer. The simultaneous treatments of *S.* Typhimurium LPS and the flavonoid compounds showed no alteration in FD4 penetration compared to untreated cells. These results highlight that Q, QM, and R are promising substances that can be used to protect intestinal epithelial cells from the deteriorating effects of oxidative stress.

## 1. Introduction

The intestinal epithelium of pigs in large-scale farming is continuously challenged–inter alia–with mycotoxicosis, weaning stress, and endotoxin-induced stress [1,2,3]. These negative effects can lead to further problems such as inflammation or oxidative stress in the intestinal tissues, contributing to the overproduction of proinflammatory cytokines (e.g., IL-6, IL-8, TNF-α) and reactive oxygen species [4]. Inflammation and oxidative stress can cause the damage of the intestinal epithelium, which can lead to the entry and spread of pathogens. Intestinal diseases related to the abovementioned factors can cause significant economic losses in livestock industry; therefore, it is essential to prevent these harmful impacts. In addition, pigs can serve as reservoirs for food-borne pathogens such as *Escherichia coli* and *Salmonella* spp. These pathogens affect millions of people every year, sometimes with severe and fatal outcomes. Thus, there is a growing demand for alternatives that can maintain pig gastrointestinal health and prevent diseases caused by environmental stress.

**Figure 1:**
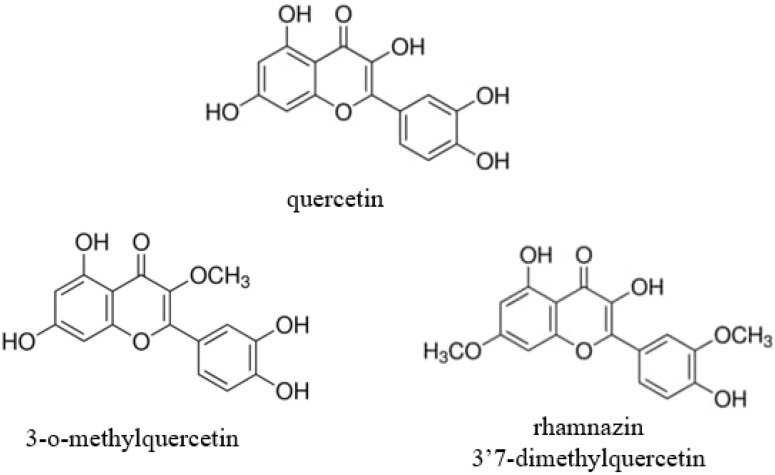
Structures of quercetin, 3-o-methylquercetin, and rhamnazin (3′7-dimethylquercetin).

Flavonoids are a large class of naturally occurring substances containing a heterocyclic pyran or pyrone ring and two benzene rings [5]. Quercetin (Q) is one of the most abundant flavanol that can be found in numerous vegetables and fruits such as onions, apples, berries, capers, dill, and also in red wine and tea [6]. Rhamnazin or 3′7-dimethylquercetin (R) and 3-o-methylquercetin (QM) (Figure 1) can also be isolated from different plants and herbs, e.g., *Viscum coloratum*, *Sarcocornia fruticosa*, *Nasturium officinale Achyrocline satureioides*, or *Semecarpus anacardium* [7,8,9,10,11]; in addition, their artificial synthesis is also available and used to produce pure forms of these substances [12,13]. Q has shown many beneficial effects, such as antioxidant, anti-inflammatory, antiviral, antibacterial, and antiprotozoal activity [5], however, its methylated derivatives are hardly investigated. The antioxidant activity of quercetin can manifest through different pathways. It can directly affect glutathione (GSH) levels by inducing GHS production, and can influence enzymatic activity by inhibiting acetylcholinesterase and butyrylcholinesterase, and enhancing the expression of catalase, GSH peroxidase, and superoxide dismutase. Furthermore, quercetin can modulate signaling pathways such as nuclear factor kappa B (NF-κB), AMP-activated protein kinase (AMPK), and mitogen-activated protein kinase (MAPK), resulting in enhanced antioxidant properties [6]. In addition, Q can directly scavenge free radicals via a reaction with hydroxyl (-OH) groups. The scavenging capacity of flavonoids is correlated to the number of free OH groups [14], however, the methylated Q derivatives could also show potent antioxidant activity by modulating several key enzymes. The antioxidant activity of rhamnazin (R) was investigated in human leukocytes and rat liver microsomes. In these in vitro studies, R showed strong antioxidant effects [15,16]. In animal experiments, it was proven that R could provide protection against lipopolysaccharide-induced acute lung injury [17]. In vitro examinations of 3-o-methylquercetin (QM) showed potent antioxidant activity and antiproliferative properties of the flavonoid on BV-2 and HepG2 cells [18,19]. QM also has strong antibacterial activity against different bacterial species such as *Escherichia coli*, *Bacillus subtilis,* and *Staphylococcus aureus* [20]. The anti-inflammatory activity of QM was also investigated in RAW 246.7 cells and the tracheal tissue of guinea pigs [21,22]. These studies reported that QM has a bronchodilator effect, suppresses total inflammatory cells, and attenuates the secretion of interleukins (IL-4, IL-5) and cytokines (TNF-α).

IPEC-J2 cells are intestinal columnar epithelial cells of mid-jejunum that are isolated from neonatal unsuckled piglets [23]. This noncancerous cell line more closely mimics the human physiology than other tumorigenic or transformed cell lines. Therefore, IPEC-J2 cells are a useful tool with which to investigate the status of the antioxidant defense system, inflammation, or epithelial integrity [24,25,26,27]. These intestinal cells express and produce different inflammatory interleukins (e.g., IL-6, IL-8), cytokines (e.g., granulocyte macrophage colony stimulating factor, TNF-α), defensins, toll-like receptors, and mucins [28,29]. Culturing on 0.4 µm pore-size membrane filters, these cells become polarized due to tight junctions and a confluent monolayer formation. Owing to these properties, the IPEC-J2 cell line is an ideal tool to investigate epithelial transport and permeability.

In this study, we examined the anti-inflammatory and antioxidant activity of Q, QM, and R in an IPEC-J2 cell line under different bacterial (*Salmonella enterica* ser. Typhimurium, *Escherichia coli* O111:B4, and *E. coli* O127:B8) LPS challenge. Furthermore, we observed the impact of these substances on the intestinal paracellular permeability. To our knowledge, the present study is the first in which the anti-inflammatory, antioxidant, and epithelial barrier protective activity of methylated quercetin derivatives was examined on healthy swine intestinal epithelial cells. Based on their structural properties and literature data in connection with inflammatory and antibacterial properties, the two derivatives seem promising compounds in preventing intestinal inflammation and oxidative stress caused by pathogenic enteric bacteria such as *E. coli* and *Salmonella* spp.

## 2. Materials and Methods

### 2.1. Chemicals

Quercetin (≥95%, HPLC grade) (Q), 3-o-methyl quercetin (≥97%, HPLC grade) (QM), rhamnazin (≥99%, HPLC grade) (R), lipopolysaccharides (LPS) (derived from *Salmonella enterica* ser. Typhimurium, *Escherichia coli* O111:B4, and *E. coli* O127:B8, suitable for cell culture) and dimethyl-sulfoxide (DMSO, Hybri-Max, sterile filtered, BioReagent, suitable for hybridoma, (≥99.7%) were purchased from Sigma-Aldrich—Merck (Darmstadt, Germany).

### 2.2. Cell Line and Culture Conditions

The IPEC-J2 cell line was a kind gift of Dr. Judy Gookin’s Department of Clinical Sciences, College of Veterinary Medicine, North Carolina State University, Raleigh, NC, USA. The origin of this cell line is the jejunum of a healthy neonatal piglet [22]. The cell culture was grown in the 1:1 mixture of Dulbecco’s Modified Eagle’s Medium and Ham’s F-12 Nutrient Mixture (DMEM/F12) (plain medium). The plain medium was supplemented with 5 ng/mL EGF, 1% penicillin streptomycin, 5 µg/mL insulin, 5 µg/mL transferrin, 5 ng/mL selenium (ITS), and 5% fetal bovine serum (FBS) (Sigma-Aldrich—Merck) for culturing. IPEC-J2 cells were grown at 37 °C in a humidified atmosphere of 5% CO_2_. For the experiments, the IPEC-J2 cells were seeded onto different cell culture plates (Corning Inc., Corning, NY, USA) between passages 48 and 52, at a density of 1.5 × 10^5^ cells/mL. The supplemented culture medium was changed every second day until confluence was achieved [28].

### 2.3. Cell Viability Measurement by the Neutral Red Uptake Assay

The influence of Q, QM, and R on the viability of IPEC-J2 cells at different concentrations (25 µM and 50 µM) was tested. Q, QM, and R were dissolved in sterile dimethyl sulfoxide (DMSO) (Sigma-Aldrich—Merck, Darmstadt, Germany). Then, stock solutions (5%) were made with plain cell culture medium, which were diluted further to the required concentrations. IPEC-J2 cells were seeded on a 96-well plate and incubated with Q, QM, and R for a 1 h treatment period. The incubation time (1 h) was based on our earlier work [27]. The percentage of living cells was determined 24 h after incubation by Neutral Red assay following the method of Repetto et al. [30].

### 2.4. Treatment of Enterocytes with Q, QM, R, and LPS

After IPEC-J2 cells reached confluency, they were rinsed twice with plain medium. LPS derived from *Salmonella enterica* ser. Typhimurium, *Escherichia coli* O111:B4, and *E. coli* O127:B8 was used to induce oxidative stress and inflammation. Control samples were treated with DMEM/F12 plain medium. LPS solutions were added to the plain medium at 10 µg/mL concentration [25]. Q, QM, and R solutions were dissolved in sterile DMSO; thereafter, stock solutions (5%) were made with plain medium, which were diluted with plain medium to reach 25 µM and 50 µM concentrations of the flavonoids. After 1 h incubation with LPS, Q, QM, and R test compounds–and their combinations–cells were washed with plain medium and cultured with plain medium for additional 6 and 24 h for inflammatory studies and redox status measurements.

### 2.5. Determination of the Amount of Intracellular Reactive Oxygen Species (ROS) and Extracellular H_2_O_2_ Levels in IPEC-J2 Cells

IPEC-J2 cells were treated with three different bacterial (*Salmonella enterica* ser. Typhimurium, *Escherichia coli* O111:B4, and *E. coli* O127:B8) LPS (10 µg/mL), Q, QM, and R (50 µM), and their combinations in phenol red-free DMEM/F12 plain medium on 24-well culture plates for 1 h. After 1 h incubation with all test compounds and their combinations, cells were washed with plain medium and cultured with plain medium for additional 24 h for redox states measurements. Extracellular H_2_O_2_ measurement was carried out by Amplex Red Hydrogen Peroxide/Peroxidase Assay Kit (Thermo Fisher Scientific, Waltham, MA, USA) following the manufacturer’s instruction [31]. Fluorescence intensity was measured at an excitation wavelength of 560 nm and an emission wavelength at 590 nm (Victor X2 2030 fluorometer, PerkinElmer, Waltham, MA, USA). The intracellular redox status of the IPEC-J2 cells was determined using a 2′,7′-dichloro-dihydro-fluorescein diacetate (DCFH-DA) dye (Sigma-Aldrich—Merck, Darmstadt, Germany). Intracellular ROS oxidize nonfluorescent DCFH-DA to fluorescent dichlorofluorescein form (DCF) [32]. DCFH-DA (10 µM) was pipetted to IPEC-J2 cells for 30 min. Cells were washed with medium, scraped (each well for 30 s), and centrifuged for 10 min at 4500 rpm at 4 °C. Fluorescence was determined with a Victor X2 2030 fluorometer at an excitation wavelength of 480 nm and an emission wavelength of 530 nm.

### 2.6. Measurement of IL-6 Levels

IPEC-J2 cells were treated with the three LPS types (10 µg/mL)–Q, QM, and R (50 µM)–and their combinations in plain cell culture medium on 24-well culture plates for 1 h. After the treatment, treatment solutions were removed, and plain cell culture medium was added to the enterocytes. Cell culture supernatants were collected 6 h after treatment [26], and IL-6 concentrations were determined from 100 µL of samples. Each sample was measured twice. IL-6 level (pg/mL) was measured with porcine-specific IL-6 ELISA kits (Sigma-Aldrich—Merck, Darmstadt, Germany) following the manufacturer’s guide.

### 2.7. Paracellular Permeability Measurement

IPEC-J2 cells were seeded on 6-well, 0.4 µm pore-size polyester membrane inserts and were grown to confluent, differentiated monolayers. Transepithelial electrical resistance (TEER) measurement of monolayers was performed on alternate days after seeding, from day 7 of culture, using an EVOM Epithelial Tissue Volt/Ohmmeter (World Precision Instruments, Berlin, Germany) [28,29]. *S*. Typhimurium LPS was added at 10 µg/mL concentration alone and with combinations of Q, QM, and R in two concentrations (25 µM and 50 µM). At the same time as LPS and combination treatments administration, 1 mg/mL fluorescein isothiocyanate dextran 4 kDa (FD4) tracer dye was added to the cells (Sigma-Aldrich, Darmstadt, Germany) with different incubation times (2 and 4 h). Supernatants from the basolateral chambers were collected, and the FD4 concentration was measured by a fluorescent method at excitation 485 nm and emission 535 nm (Perkin Elmer, Victor X2 2030 fluorimeter).

### 2.8. Statistics

Statistical analysis of our data was performed with R 3.3.2 (2016) software (R Foundation, Vienna, Austria). Differences between means were determined by two-way ANOVA, with data of normal distribution, and homogeneity of variances was also confirmed. The Dunnett post hoc test was applied to analyze treated groups to in comparison to control, while the Fisher LSD test was used to compare different treatments. Differences between groups were considered proven if *p* values were <0.05.

## 3. Results

### 3.1. IPEC-J2 Cell Viability

**Figure 2:**
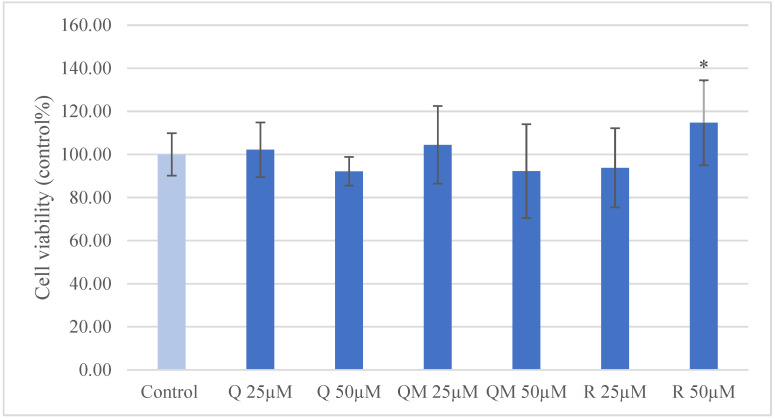
Viability of porcine intestinal epithelial cells (IPEC-J2) after treatment with different concentrations (25 µM and 50 µM) of quercetin (Q), 3-o-methylquercetin (QM) and rhamnazin (R). Data are shown as means with standard deviations (*n* = 6/group; * *p* < 0.05) in control percentage.

To determine the proper concentration of Q, QM, and R for the investigations without cell viability reduction, we used Neutral red uptake assay. Using the 25 µM and 50 µM concentrations, there was no significant reduction of cell viability; moreover, the higher concentration of R increased the quantity of living enterocytes compared to the control group (Figure 2).

### 3.2. Production of ROS in IPEC-J2 Cells after Treatment of Q, QM, and R

**Figure 3:**
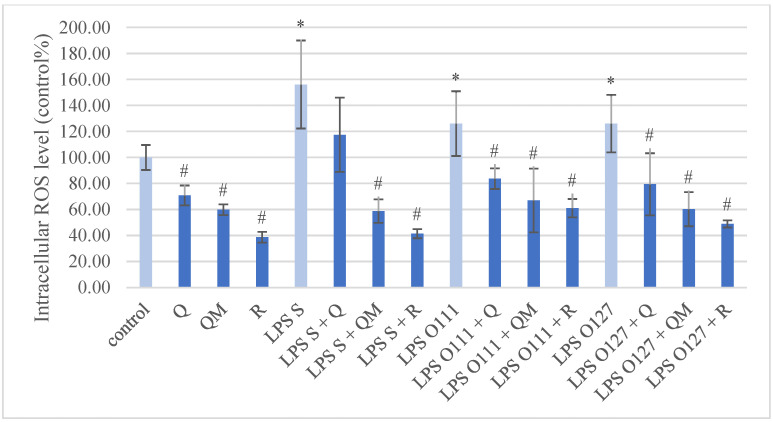
Intracellular reactive oxygen species level in porcine intestinal epithelial cells (IPEC-J2) after treatment with different types of lipopolysaccharides (LPS) from *Salmonella* Typhimurium (LPS S), *E. coli* O111:B4 (LPS O111), and *E. coli* O127:B8 (LPS O127) in 10 µg/mL concentration; quercetin (Q), 3-o-methylquercetin (QM) and rhamnazin (R) in 50 µM concentration; and their combinations. Data are shown as means with standard deviations (*n* = 6/group; * significantly higher *p* < 0.05; # significantly lower *p* < 0.05) in control percentage and are statistically compared to those of the control group.

Compared to the control group, the intracellular ROS level of IPEC-J2 cells showed a significant increase after all types of LPS stimulation (Figure 3). Q, QM, and R per se in 50 µM concentration significantly decreased the intracellular ROS quantity compared to the control. All simultaneous treatment of LPS strains and quercetin derivatives significantly decreased the intracellular ROS level, except the Q and *S*. Typhimurium LPS combination.

**Figure 4:**
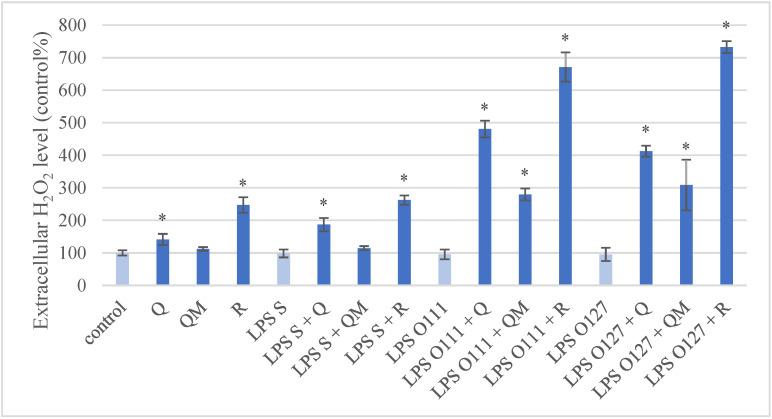
Extracellular hydrogen peroxide (H_2_O_2_) level in porcine intestinal epithelial cells (IPEC-J2) after treatment with different types of lipopolysaccharides (LPS) from *Salmonella* Typhimurium (LPS S), *E. coli* O55:B5 (LPS O55), *E. coli* O111:B4 (LPS O111), and *E. coli* O127:B8 (LPS O127) in 10 µg/mL concentration; quercetin (Q), 3-o-methylquercetin (QM) and rhamnazin (R) in 50 µM concentration; and their combinations. Data are shown as means with standard deviations (*n* = 6/group; * *p* < 0.05) in control percentage and are statistically compared to those of the control group.

Using the Amplex Red Hydrogen Peroxide/Peroxidase Assay Kit, the extracellular H_2_O_2_ level showed a significant increase after the Q and R (50 µM) treatments compared to the control group (Figure 4). With a similar comparison, there was no significant alteration in H_2_O_2_ quantity after the different types of LPS treatment. However, all the simultaneous treatments increased the H_2_O_2_ level significantly, except for the QM and *S*. Typhimurium LPS combination. QM per se did not cause a significant change compared to the control group.

### 3.3. Production of IL-6 in IPEC-J2 Cells after Treatment of Q, QM and R

**Figure 5:**
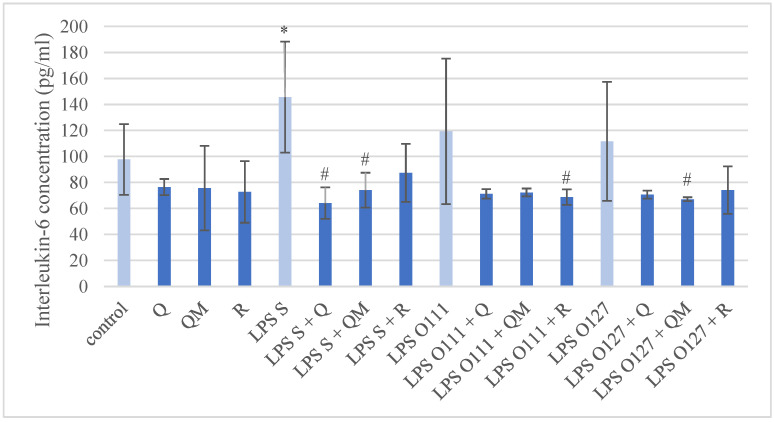
Interleukin-6 (IL-6) concentration in porcine intestinal epithelial cells (IPEC-J2) after treatment with different types of lipopolysaccharides (LPS) from *Salmonella* Typhimurium (LPS S), *E. coli* O111:B4 (LPS O111), and *E. coli* O127:B8 (LPS O127) in 10 µg/mL concentration; quercetin (Q), 3-o-methylquercetin (QM) and rhamnazin (R) in 50 µM concentration; and their combinations. Data are shown as means with standard deviation (*n* = 6/group; * significantly higher *p* < 0.05; # significantly lower *p* < 0.05) and are statistically compared to those of the control group.

The IL-6 production of IPEC-J2 cells is shown in Figure 5. The *S*. Typhimurium LPS caused significant elevation in IL-6 production compared to the control group, however, the LPS types derived from different *E. coli* strains did not evoke a significantly increased IL-6 concentration.

In similar comparison, all the quercetin derivatives in 50 µM concentration did not influence the IL-6 production per se. The simultaneous treatments of *S*. Typhimurium LPS with Q or QM significantly decreased the IL-6 concentration, and likewise for *E. coli* O111:B4 LPS with R, and *E. coli* O127:B8 LPS with QM.

### 3.4. Paracellular Permeability of IPEC-J2 Cells after Treatment of Q, QM, and R

**Figure 6:**
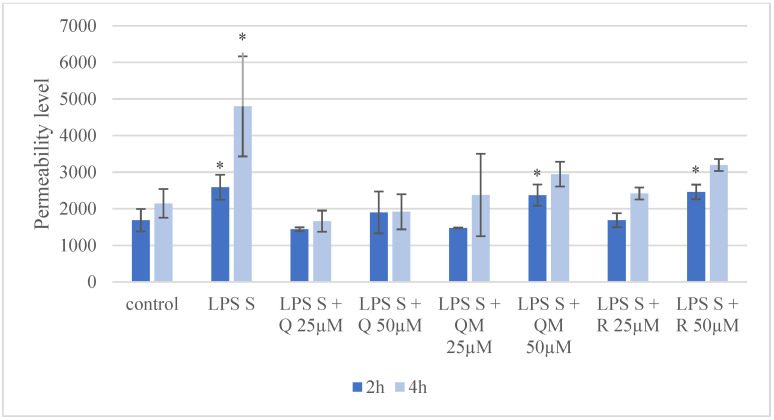
Penetration of FD4 from the apical to the basolateral compartment through IPEC-J2 cells after *Salmonella* Typhimurium LPS (LPS S) treatment (10 µg/mL, treatment time 1 h, detection after 2 h and 4 h) and with combinations of quercetin (Q), 3-o-methylquercetin (QM) and rhamnazin (R) using two concentrations (25 µM and 50 µM). Data are shown as means with standard deviations (*n* = 3/group; * *p* < 0.05) and are statistically compared to those of the control group.

Partial disruption of the epithelial monolayer was investigated following 2 and 4 h after *S*. Typhimurium LPS treatment (Figure 6). Compared to the untreated samples, the fluorescence intensity of FD4 in the basolateral chamber was significantly higher after LPS treatment at both time appointments. The lower concentration (25 µM) of Q, QM, and R combined with LPS did not cause significant alteration in fluorescence intensity of FD4 at both time appointments. The simultaneous treatments of LPS with QM and R in 50 µM concentration at 2 h significantly increased the presence of the FD4 tracer compared to the control group, however it was not observed at 4 h.

## 4. Discussion

In order to maintain economical production in swine industry, negative environmental impacts such as oxidative stress or inflammation should be avoided or prevented. Since the One Health Approach came into view recently, antimicrobial usage has had strict restrictions (Regulation EU 2019/6) to adhere to in order to preserve their efficiency both in human and veterinary practice; therefore, there is a growing interest to replace antibiotics with natural alternatives. Quercetin, 3-o-methylquercetin, and rhamnazin are promising naturally occurring substances due to their beneficial properties such as antioxidant, anti-inflammatory, and antibacterial activity. The bioavailability and transport of flavonoids depend on several factors. Methylated flavonoids are reported to have better bioavailability and stability [33]. Quercetin glucosides have shown lower penetration in a Caco-2 cell line model [34] compared to aglycon form, while methylated flavones could reach higher penetration through a Caco-2 cell layer [33]. Due to extensive metabolism in the intestine, the oral bioavailability of quercetin aglycon is close to zero in humans [35], calves [36], and pigs [37]. Maciej et al. compared quercetin aglycon and rutin (quercetin rutinoside) in a 9 mg/kg oral dose and found that aglycone had better absorption. Ader et al. concluded that the oral bioavailability of quercetin in pigs after 50 mg/kg dose is 0.54%, which could be enhanced (8.6%) with glucuronic and sulfuric acid conjugation. In addition, the biological activity of quercetin can be different from unmethylated ones, therefore, our aim was to investigate the differences in Q, QM, and R regarding their antioxidant, anti-inflammatory, and epithelial barrier protecting activity.

The intestinal wall is one of the first protecting layer against pathogenic enteric bacteria and toxins, therefore it is important to keep its appropriate function and integrity. The IPEC-J2 cell line is a suitable tool to model healthy intestinal epithelial layer in pigs. In pigs, gastrointestinal infections caused by *E. coli* and *Salmonella* spp. are of high importance, as both pathogens are widespread, potentially zoonotic, and highly prone to developing resistance. Therefore, there is a need for an in vitro tool to study the gastrointestinal tract and diseases of the pigs. Furthermore, due to the anatomic and physiologic similarities between swine and human, this cell line is widely used as a human in vitro model [29]. Due to the close similarity between swine and human intestinal function, studies with IPEC-J2 cells provide valuable insights into the pathogenesis of zoonotic enteric infections that also affect humans [38]. Overall, the relevance of the IPEC-J2 cell line can be equally important from a human and veterinary point of view.

Among other antioxidant compounds, Q was examined with Neutral red assay in different concentrations (6.25–800 µM) using IPEC-J2 cells [39]. Concentrations between 6.25–200 µM Q significantly increased cell viability, 400 µM Q did not caused alteration, and 800 µM Q significantly decreased cell viability. In contrast, our study demonstrated that the treatments of Q (25 and 50 µM) did not increase cell viability. This can be explained with the treatment period. Vergauwen et al. (2016) used 18 h pretreatment [39], while we used 1 h. We ascertained that 50 µM R treatment significantly improved cell viability; QM in both applied (25 and 50 µM) and R in the lower concentration (25 µM) did not lead to cell death.

Inflammation and oxidative stress in IPEC-J2 cells can be provoked with different types of LPS such as *Salmonella enterica* serovar Typhimurium and serovar Choleraesuis, enterotoxigenic *Escherichia coli* (K88), or *E. coli* L2880 [26,40,41,42,43]. Our results confirm that *S*. Typhimurium-derived LPS can increase intracellular ROS level and IL-6 concentration in porcine intestinal epithelial cells. There are numerous in vitro and in vivo investigations involving the antioxidant effect of Q in different cell types, which are summarized by Xu et al. [6], although its antioxidant activity on IPEC-J2 cells after LPS challenge has not been observed till now. A recent study described that Q could attenuate diquat-induced oxidative stress in porcine intestinal enterocytes by regulating GSH-related homeostasis and elevating protein abundance of nuclear factor erythroid-2 [44]. Our results showed that Q and its methylated derivatives could decrease intracellular ROS quantity in porcine intestinal enterocytes after LPS challenge. Furthermore, the methylated derivatives (QM and R) were considered as more effective antioxidants than Q in the case of *S*. Typhimurium LPS-induced oxidative stress. In contrast, the extracellular H_2_O_2_ level was increased after Q and R treatment with or without LPS treatment, which indicates that these flavonoids can have pro-oxidant properties as well; this can be explained by their different structure. Kessler et al. found that the hydroxyl group on the C3 position of Q increases the pro-oxidant activity, while the methylation on C7 enhances the scavenging activity [45]. On the other hand, Duenas et al. concluded that the methylation of C3′ and C4′ positions of Q decrease the scavenging activity [46].

The anti-inflammatory effect of Q was described among others in IEC-6 cells [47], Caco-2 cells [48], RAW264.7 cells [49], and lung epithelial cells [50]. Cai et al. described that Q (5 µmol/L) can decrease the production of IL-1β, IL-6, TNF-α and prostaglandin E2 (PGE2) after LPS stimulation. In Caco-2 cells, Han et al. observed similar anti-inflammatory activity with 5, 10, and 20 µM Q treatments. In their study, Q decreased cyclooxygenase-2, TNF-α, and IL-1β production. Tang et al. and Sul and Ra noted that Q can decrease IL-6 and TNF-α concentration after LPS induced inflammation. There are fewer studies about methylated Q derivatives, however, Ren et al. concluded that a methylated quercetin (isorhamnetin; 3′-o-methylquercetin) can inhibit TNF-α induced inflammation in human bronchial epithelia [51]. Novo Belchor et al. compared the anti-inflammatory activity of Q with methylated derivatives (3-o-methylquercetin, rhamnetin and rhamnazin) [52]. They found that rhamnetin (7-o-methylquercetin) has the most potent anti-inflammatory activity through the inhibition of secretory phospholipases A2 enzymes. Another flavonoid–chrysin–and its dimethylated form were compared regarding their anti-inflammatory activity in Caco-2 cells [53]. The results of this study showed that methylated chrysin had more potent activity to decrease IL-6 and PGE2 production. Our results highlighted that Q (50 µM) significantly decrease IL-6 production in IPEC-J2 cells; moreover, we described first that QM (50 µM) has the same effect in IPEC-J2 cells.

The intestinal epithelial barrier integrity of IPEC-J2 cells was studied after LPS-induced inflammation in several investigations. Zhao et al. examined the protective effect of a phytochemical (capsaicin) on the cell layer integrity of IPEC-J2 cells after LPS challenge [54]. They found that capsaicin attenuates LPS induced inflammation and improves barrier integrity. On the other hand, Jiang et al. (2021) disrupted the barrier function of IPEC-J2 cells with LPS and deoxynivalenol, which could not be alleviated with 4-phenylbutyric acid [55]. These investigations correlated with our results that LPS can increase the permeability of FD4 fluorescent tracer. Vergauwen et al. (2016) used 1 mM H_2_O_2_ to disrupt the IPEC-J2 cell monolayer [39]. In this study, quercetin was used in 25, 50, 100, and 200 µM concentrations to preserve the cell layer integrity, and it was concluded that all treatments significantly decreased the FD4 penetration. In our investigation we found that after four hours, all the tested substances (Q, QM, and R) could alleviate the disruption of epithelial layer after being challenged by LPS.

## 5. Conclusions

This study demonstrated that Q, QM, and R have beneficial effects on healthy porcine intestinal epithelial cells. To our knowledge, this is the first time that QM and R have been examined on IPEC-J2 cells and that their antioxidant and anti-inflammatory effect has been confirmed. None of the tested compounds caused cell death in 25 µM and 50 µM concentrations. Both Q and its methylated derivatives could decrease intracellular ROS production in LPS-evoked oxidative stress. Furthermore, in 25 µM concentration, all quercetin types prevented the disruption of confluent intestinal epithelial monolayer damaged with *S*. Typhimurium LPS. In terms of our results, Q, QM, and R are promising substances to prevent or treat intestinal impairments caused by pathogenic enteral bacteria in the swine industry and in human medicine. However, the bioavailability of these substances could be very diverse and depends on many factors; thus, further in vitro and in vivo investigations are required to utilize their advantages.

## Data Availability

Data is contained within the article.

## References

[1] Jiang Z.Y., Sun L.H., Lin Y.C., Ma X.Y., Zheng C.T., Zhou G.L., Chen F., Zou S.T. (2009). Effects of dietary glycyl-glutamine on growth performance, small intestinal integrity, and immune responses of weaning piglets challenged with lipopolysaccharide1. J. Anim. Sci..

[2] Hu J., Chen L., Zheng W., Shi M., Liu L., Xie C., Wang X., Niu Y., Hou Q., Xu X. (2018). Lactobacillus frumenti Facilitates Intestinal Epithelial Barrier Function Maintenance in Early-Weaned Piglets. Front. Microbiol..

[3] Chen Z., Yuan Q., Xu G., Chen H., Lei H., Su J. (2018). Effects of Quercetin on Proliferation and H2O2-Induced Apoptosis of Intestinal Porcine Enterocyte Cells. Molecules.

[4] Abreu M.T. (2010). Toll-like receptor signalling in the intestinal epithelium: How bacterial recognition shapes intestinal function. Nat. Rev. Immunol..

[5] Batiha G.E.-S., Beshbishy A.M., Ikram M., Mulla Z.S., El-Hack M.E.A., Taha A.E., Algammal A.M., Elewa Y.H.A. (2020). The Pharmacological Activity, Biochemical Properties, and Pharmacokinetics of the Major Natural Polyphenolic Flavonoid: Quercetin. Foods.

[6] Xu D., Hu M.-J., Wang Y.-Q., Cui Y.-L. (2019). Antioxidant Activities of Quercetin and Its Complexes for Medicinal Application. Molecules.

[7] Zhao Y., Yu Z., Fan R., Gao X., Yu M., Li H., Wei H., Bi K. (2011). Simultaneous Determination of Ten Flavonoids from Viscum coloratum Grown on Different Host Species and Different Sources by LC-MS. Chem. Pharm. Bull..

[8] Hawas U.W., El-Kassem L.T.A., Shaher F., Al-Farawati R. (2018). In vitro inhibition of Hepatitis C virus protease and antioxidant by flavonoid glycosides from the Saudi costal plant *Sarcocornia fruticosa*. Nat. Prod. Res..

[9] Goda Y., Hoshino K., Akiyama H., Ishikawa T., Abe Y., Nakamura T., Otsuka H., Takeda Y., Tanimura A., Toyoda M. (1999). Constituents in Watercress: Inhibitors of Histamine Release from RBL-2H3 Cells Induced by Antigen Stimulation. Biol. Pharm. Bull..

[10] Bianchi S.E., Kaiser S., Pittol V., Doneda E., De Souza K.C.B., Bassani V.L. (2018). Semi-preparative isolation and purification of phenolic compounds from *Achyrocline satureioides* (Lam) D.C. by high-performance counter-current chromatography. Phytochem. Anal..

[11] Kumar A.D.N., Bevara G.B., Kaja L.K., Badana A.K., Malla R.R. (2016). Protective effect of 3-*O*-methyl quercetin and kaempferol from Semecarpus anacardium against H_2_O_2_ induced cytotoxicity in lung and liver cells. BMC Complement. Altern. Med..

[12] Boers F., Deng B.-L., Lemière G., Lepoivre J., De Groot A., Dommisse R., Vlietinck A.J. (1997). An Improved Synthesis of the Anti-Picornavirus Flavone 3-O-Methylquercetin. Arch. der Pharm..

[13] Rao K.V., Seshadri T.R. (1946). Synthesis of rhamnazin. J. Chem. Soc..

[14] Lesjak M., Beara I., Simin N., Pintać D., Majkić T., Bekvalac K., Orčić D., Mimica-Dukić N. (2018). Antioxidant and anti-inflammatory activities of quercetin and its derivatives. J. Funct. Foods.

[15] Yun B.-S., Lee I.-K., Kim J.-P., Chung S.-H., Shim G.-S., Yoo I.-D. (2000). Lipid peroxidation inhibitory activity of some constituents isolated from the stem bark ofEucalyptus globulus. Arch. Pharmacal Res..

[16] Martini N., Katerere D., Eloff J. (2004). Biological activity of five antibacterial flavonoids from Combretum erythrophyllum (Combretaceae). J. Ethnopharmacol..

[17] Wu G., Dai X., Li X., Jiang H. (2017). Antioxidant and Anti-Inflammatory Effects of Rhamnazin on Lipopolysaccharide-Induced Acute Lung Injury and Inflammation in Rats. Afr. J. Tradit. Complement. Altern. Med..

[18] Kim J.Y., Lim H.J., Ryu J.-H. (2008). In vitro anti-inflammatory activity of 3-*O*-methyl-flavones isolated from Siegesbeckia glabrescens. Bioorg. Med. Chem. Lett..

[19] Zhang Q., Yang W., Liu J., Liu H., Lv Z., Zhang C., Chen D., Jiao Z. (2020). Identification of Six Flavonoids as Novel Cellular Antioxidants and Their Structure-Activity Relationship. Oxidative Med. Cell. Longev..

[20] Wang J., Lou J., Luo C., Zhou L., Wang M., Wang L. (2012). Phenolic Compounds from *Halimodendron halodendron* (Pall.) Voss and Their Antimicrobial and Antioxidant Activities. Int. J. Mol. Sci..

[21] Ko W.-C., Shih C.-M., Chen M.-C., Lai Y.-H., Chen J.-H., Chen C.-M., Lin C.-N. (2004). Suppressive Effects of 3-*O*-Methylquercetin on Ovalbumin-Induced Airway Hyperresponsiveness. Planta Med..

[22] Jiang J.-S., Shih C.-M., Wang S.-H., Chen T.-T., Lin C.-N., Ko W.-C. (2006). Mechanisms of suppression of nitric oxide production by 3-O-methylquercetin in RAW 264.7 cells. J. Ethnopharmacol..

[23] Brosnahan A.J., Brown D.R. (2012). Porcine IPEC-J2 intestinal epithelial cells in microbiological investigations. Vet. Microbiol..

[24] Farkas O., Mátis G., Pászti-Gere E., Palócz O., Kulcsár A., Petrilla J., Csikó G., Neogrády Z., Gálfi P. (2014). Effects of Lactobacillus plantarum 2142 and sodium n-butyrate in lipopolysaccharide-triggered inflammation: Comparison of a porcine intestinal epithelial cell line and primary hepatocyte monocultures with a porcine enterohepatic co-culture system12. J. Anim. Sci..

[25] Farkas O., Palócz O., Pászti-Gere E., Gálfi P. (2015). Polymethoxyflavone Apigenin-Trimethylether Suppresses LPS-Induced Inflammatory Response in Nontransformed Porcine Intestinal Cell Line IPEC-J2. Oxidative Med. Cell. Longev..

[26] Palócz O., Pászti-Gere E., Gálfi P., Farkas O. (2016). Chlorogenic Acid Combined with Lactobacillus plantarum 2142 Reduced LPS-Induced Intestinal Inflammation and Oxidative Stress in IPEC-J2 Cells. PLoS ONE.

[27] Karancsi Z., Móritz A.V., Lewin N., Veres A.M., Farkas O. (2020). Beneficial Effect of a Fermented Wheat Germ Extract in Intestinal Epithelial Cells in case of Lipopolysaccharide-Evoked Inflammation. Oxidative Med. Cell. Longev..

[28] Schierack P., Nordhoff M., Pollmann M., Weyrauch K.D., Amasheh S., Lodemann U., Jores J., Tachu B., Kleta S., Blikslager A. (2005). Characterization of a porcine intestinal epithelial cell line for in vitro studies of microbial pathogenesis in swine. Histochemistry.

[29] Vergauwen H., Verhoeckx K., Cotter P., López-Expósito I., Kleiveland C., Lea T., Mackie A., Requena T., Swiatecka D., Wichers H. (2015). The IPEC-J2 Cell Line. The Impact of Food Bioactives on Health: In Vitro and Ex Vivo Models.

[30] Repetto G., del Peso A., Zurita J.L. (2008). Neutral red uptake assay for the estimation of cell viability/cytotoxicity. Nat. Protoc..

[31] Mohanty J., Jaffe J.S., Schulman E.S., Raible D.G. (1997). A highly sensitive fluorescent micro-assay of H2O2 release from activated human leukocytes using a dihydroxyphenoxazine derivative. J. Immunol. Methods.

[32] Wang H., Joseph J.A. (1999). Quantifying cellular oxidative stress by dichlorofluorescein assay using microplate reader. Free Radic. Biol. Med..

[33] Wen X., Walle T. (2006). Methylated Flavonoids Have Greatly Improved Intestinal Absorption and Metabolic Stability. Drug Metab. Dispos..

[34] Walgren A.R., Walle U., Walle T. (1998). Transport of Quercetin and Its Glucosides across Human Intestinal Epithelial Caco-2 Cells. Biochem. Pharmacol..

[35] Walle T. (2004). Absorption and metabolism of flavonoids. Free Radic. Biol. Med..

[36] Maciej J., Schäff C., Kanitz E., Tuchscherer A., Bruckmaier R., Wolffram S., Hammon H. (2015). Bioavailability of the flavonol quercetin in neonatal calves after oral administration of quercetin aglycone or rutin. J. Dairy Sci..

[37] Ader P. (2000). Bioavailability and metabolism of the flavonol quercetin in the pig. Free Radic. Biol. Med..

[38] Skjolaas K., Burkey T., Dritz S., Minton J. (2006). Effects of Salmonella enterica serovars Typhimurium (ST) and Choleraesuis (SC) on chemokine and cytokine expression in swine ileum and jejunal epithelial cells. Veter-Immunol. Immunopathol..

[39] Vergauwen H., Prims S., DeGroote J., Wang W., Casteleyn C., Van Cruchten S., De Smet S., Michiels J., Van Ginneken C. (2016). In Vitro Investigation of Six Antioxidants for Pig Diets. Antioxidants.

[40] Burkey T., Skjolaas K., Dritz S., Minton J. (2007). Expression of Toll-like receptors, interleukin 8, macrophage migration inhibitory factor, and osteopontin in tissues from pigs challenged with Salmonella enterica serovar Typhimurium or serovar Choleraesuis. Veter-Immunol. Immunopathol..

[41] Devriendt B., Stuyven E., Verdonck F., Goddeeris B., Cox E. (2010). Enterotoxigenic Escherichia coli (K88) induce proinflammatory responses in porcine intestinal epithelial cells. Dev. Comp. Immunol..

[42] Kovács D., Karancsi Z., Farkas O., Jerzsele Á. (2020). Antioxidant Activity of Flavonoids in LPS-Treated IPEC-J2 Porcine Intestinal Epithelial Cells and Their Antibacterial Effect against Bacteria of Swine Origin. Antioxidants.

[43] Bao M., Liang M., Sun X., Mohyuddin S.G., Chen S., Wen J., Yong Y., Ma X., Yu Z., Ju X. (2022). Baicalin Alleviates LPS-Induced Oxidative Stress via NF-κB and Nrf2–HO1 Signaling Pathways in IPEC-J2 Cells. Front. Vet. Sci..

[44] Jia H., Zhang Y., Si X., Jin Y., Jiang D., Dai Z., Wu Z. (2021). Quercetin Alleviates Oxidative Damage by Activating Nuclear Factor Erythroid 2-Related Factor 2 Signaling in Porcine Enterocytes. Nutrients.

[45] Kessler M., Ubeaud G., Jung L. (2003). Anti- and pro-oxidant activity of rutin and quercetin derivatives. J. Pharm. Pharmacol..

[46] Dueñas M., González-Manzano S., González-Paramás A., Santos-Buelga C. (2010). Antioxidant evaluation of O-methylated metabolites of catechin, epicatechin and quercetin. J. Pharm. Biomed. Anal..

[47] Cai S.-Q., Zhang Q., Zhao X.-H., Shi J. (2021). The In Vitro Anti-Inflammatory Activities of Galangin and Quercetin towards the LPS-Injured Rat Intestinal Epithelial (IEC-6) Cells as Affected by Heat Treatment. Molecules.

[48] Song Y., Han M., Zhang X. (2016). Quercetin suppresses the migration and invasion in human colon cancer Caco-2 cells through regulating toll-like receptor 4/Nuclear Factor-kappa B pathway. Pharmacogn. Mag..

[49] Tang J., Diao P., Shu X., Li L., Xiong L. (2019). Quercetin and Quercitrin Attenuates the Inflammatory Response and Oxidative Stress in LPS-Induced RAW264.7 Cells: In Vitro Assessment and a Theoretical Model. BioMed Res. Int..

[50] Sul O.-J., Ra S.W. (2021). Quercetin Prevents LPS-Induced Oxidative Stress and Inflammation by Modulating NOX2/ROS/NF-kB in Lung Epithelial Cells. Molecules.

[51] Ren X., Han L., Li Y., Zhao H., Zhang Z., Zhuang Y., Zhong M., Wang Q., Ma W., Wang Y. (2020). Isorhamnetin attenuates TNF -α-induced inflammation, proliferation, and migration in human bronchial epithelial cells via MAPK and NF-κB pathways. Anat. Rec..

[52] Belchor M.N., Gaeta H.H., Rodrigues C.F.B., Costa C.R.D.C., Toyama D.D.O., Passero L.F.D., Laurenti M.D., Toyama M.H. (2017). Evaluation of Rhamnetin as an Inhibitor of the Pharmacological Effect of Secretory Phospholipase A2. Molecules.

[53] During A., Larondelle Y. (2013). The O-methylation of chrysin markedly improves its intestinal anti-inflammatory properties: Structure–activity relationships of flavones. Biochem. Pharmacol..

[54] Zhao X., Dong B., Friesen M., Liu S., Zhu C., Yang C. (2021). Capsaicin Attenuates Lipopolysaccharide-Induced Inflammation and Barrier Dysfunction in Intestinal Porcine Epithelial Cell Line-J2. Front. Physiol..

[55] Jiang Q., Yin J., Chen J., Ma X., Wu M., Li X., Yao K., Tan B., Yin Y. (2021). 4-Phenylbutyric acid accelerates rehabilitation of barrier function in IPEC-J2 cell monolayer model. Anim. Nutr..

